# *Aspergillus fumigatus* in Poultry

**DOI:** 10.1155/2011/746356

**Published:** 2011-06-14

**Authors:** Pascal Arné, Simon Thierry, Dongying Wang, Manjula Deville, Guillaume Le Loc'h, Anaïs Desoutter, Françoise Féménia, Adélaïde Nieguitsila, Weiyi Huang, René Chermette, Jacques Guillot

**Affiliations:** ^1^UMR BIPAR, Ecopham, Ecole Nationale Vétérinaire d'Alfort (ENVA), 94700 Maisons-Alfort, France; ^2^ANSES, UMR BIPAR, Ecopham, Ecole Nationale Vétérinaire d'Alfort, 94700 Maisons-Alfort, France; ^3^Parasitology Department, College of Animal Science and Technology, Guangxi University, Nanning, China; ^4^Parasitology-Mycology Department, Ecole Nationale Vétérinaire d'Alfort, 94700 Maisons-Alfort, France; ^5^Emirates Centre for Wildlife Propagation, Missour, Morocco; ^6^INRA, UMR BIPAR, Ecole Nationale Vétérinaire d'Alfort, 94700 Maisons-Alfort, France

## Abstract

*Aspergillus fumigatus* remains a major respiratory pathogen in birds. In poultry, infection by *A. fumigatus* may induce significant economic losses particularly in turkey production. *A. fumigatus *develops and sporulates easily in poor quality bedding or contaminated feedstuffs in indoor farm environments. Inadequate ventilation and dusty conditions increase the risk of bird exposure to aerosolized spores. Acute cases are seen in young animals following inhalation of spores, causing high morbidity and mortality. The chronic form affects older birds and looks more sporadic. The respiratory tract is the primary site of *A. fumigatus* development leading to severe respiratory distress and associated granulomatous airsacculitis and pneumonia. Treatments for infected poultry are nonexistent; therefore, prevention is the only way to protect poultry. Development of avian models of aspergillosis may improve our understanding of its pathogenesis, which remains poorly understood.

## 1. Introduction

*Aspergillus fumigatus* is considered as a major respiratory pathogen in birds. This filamentous fungus was first found in the lungs of a bustard (*Otis tarda*) in 1863 by Fresenius. Other species like *A. flavus*, *A. niger*, *A. nidulans,* and *A. terreus *may also be isolated from avian cases of aspergillosis (sometimes in mixed infections) but much less frequently than *A. fumigatus* [1–6]. Active fungal proliferation and sporulation of *A. fumigatus* on organic material produce large amounts of airborne small-sized conidia that are easily dispersed in air, then potentially inhaled and deposited deep in the respiratory tract. Susceptible hosts will develop polymorphic clinical forms in relation to either localized or disseminated lesions. Acute aspergillosis generally occurs in young birds resulting in high morbidity and mortality. The chronic form is sporadic. It causes lesser mortality and generally affects older birds, especially breeders in poultry, presenting a compromised immune system due to poor husbandry conditions [4, 7]. 

*A. fumigatus *has been isolated from lesions in wild birds since the early 1800s. Major die-offs of free-ranging wild birds have been reported from waterfowl, gulls, and corvids following dumping of mouldy waste grains in areas where birds feed [6, 8–10]. Infection by *A. fumigatus* is also found in birds of prey, penguins, and parrots held in captivity [7, 11]. Incidence may be elevated in debilitated birds sheltered in wildlife centres and severely impair rehabilitation success [12, 13].

Infection by *Aspergillus *sp. has been reported in almost all domesticated avian species and production types: layer cockerels [14], pullets in cages [15], broiler breeders [5], and growers of chicken [3, 16] or turkey poults [17–20], common duck breeders [21], goslings [1, 22], great rheas [23], ostriches [2], Japanese quails [24], or pigeons [25]. In spontaneous outbreaks, mortality ranged between 4.5% and 90%, whereas age of diseased birds varied from 3 days to 20 weeks [3–5, 14, 16–21, 26, 27]. Beside direct losses related to mortality, feed conversion and growth rate in recovering birds remain poor. Indeed, airsacculitis is a major reason for carcass condemnation at slaughter inspection [4, 28–30]. Economical significance of aspergillosis is most readily apparent in turkey production where disease occurs late in the growing cycle or primarily affects costly breeder toms [4].

## 2. Birds Exposure to *Aspergillus fumigatus* in Poultry Confinement Houses

### 2.1. Ecology of *Aspergillus fumigates* in Poultry Houses

Initial contamination of poultry farms may occur through use of a mouldy litter or introduction of one-day-old birds whose down has retained conidia in hatchery facilities. Further contamination may involve inappropriate bedding management [2, 16, 17], poor quality feedstuffs, or admission of outside air loaded in conidia [5]. Organic substrates like litter, feed, and even feathers [31] can easily fulfil nutrient requirements of *A. fumigatus* [4, 32, 33]. Humidity and temperature conditions encountered in poultry farms promote the rapid growth of hyphae and efficient asexual multiplication resulting in a copious production of easily airborne hydrophobic conidia, which are subsequently dispersed and inhaled by the birds [4, 34]. Transfers of conidia between the putative bedding reservoir [17, 34] and indoor atmosphere are still poorly understood [35]. Constant animal movements under high stocking densities, litter refreshing [17, 26, 36], or deficient ventilation [34, 35] may contribute to generate a conidial aerosol. A short-time exposure to heavily contaminated wood shavings induced an experimental pulmonary aspergillosis in chickens [37, 38] and turkeys [39]. Birds inhale the air and contact litter with continual exposure to the conidia. Therefore, a comprehensive understanding of contributory factors leading to productive infection requires precise information on instantaneous conidia concentration on one hand and fluctuations in aerosol composition on the other hand prevailing in production facilities. Numerous longitudinal surveys have been conducted in different production systems including layers [40, 41] and broilers [34, 36, 42–45] in order to characterize the mycoflora of litter, feedstuffs, and air inside the buildings. Several investigations were associated with current [17, 26] or previous [44] outbreaks of aspergillosis.

Air samples were collected either by sedimentation [41, 46], filtration [34, 42, 45], or impaction [41, 44, 45] with appropriate biocollectors. Viable and cultivable fungi were generally counted on standard agar media designed for mycological identification (Sabouraud or malt agars most frequently). Culture-independent techniques, like PCR-TTGE or PCR-D-HPLC, have been developed to monitor fungal aerosol communities in broiler farms and proved complementary with classical methods [47, 48]. 

### 2.2. Air Mycoflora

In farms, which were free from aspergillosis, the concentration of *Aspergillus *spp. in the air varied from 10 to 10^4^ CFU/m^3^ either in chicken [42, 43, 45–47] or turkey houses [35, 44]. Although up to more than sixty different species have been identified in a turkey confinement brooder house [34], a few genera, namely, *Aspergillus*, *Penicillium*, *Cladosporium*, *Fusarium*, and *Scopulariopsis,* constitute the majority of fungal isolation [35, 41–43, 45–47]. Prevalence and relative importance of *Aspergillus* species (*A. fumigatus*, *A. flavus*, *A. nidulans*, or *A. amstelodami*) can vary significantly [35, 41, 44, 46]. In fact, air contamination is characterized by cyclic variations as evidenced by weekly sampling [42, 44]. These fluctuations may be related to season or husbandry management [34, 35, 44]. In several healthy turkey flocks, air concentration of *Aspergillus *spp. measured in the winter was fifteen times higher than in summer [35]. Concentration of *Aspergillus* spp. which predominated in air and litter in a turkey farm decreased drastically when the windows were opened [34], whereas no significant quantitative differences were attributable to house ventilation design in other surveys [35, 43]. The negative correlation between relative humidity and the number of *Aspergillus* conidia in air may indicate that xerophilic *Aspergillus* conidia more readily discharge in dry conditions than in humid atmosphere. Interestingly, high counts of* A. fumigatus* conidia in air coincided with high levels of respirable dust particles suggesting a possible physical association or a similar response to indoor conditions [35]. Sawdust generated by both litter and feed, harboured numerous fungi as dormant propagules due to low moisture that could serve as an inoculum for fresh litter [36, 49, 50].

### 2.3. Mycoflora of Poultry Beddings

The biodiversity of litter mycoflora depends on material choice, litter aging, and handling techniques [49]. More than thirty different taxa were identified in wood chips of turkey facilities [34] and shavings in broiler houses with a predominance of *Aspergillus*, *Scopulariopis*, and *Penicillium *[34, 36, 40, 49, 51]. Using an immersion technique with strip baits, Bacon and Burdick [36] isolated 18 fungal species from poultry litter. The same fungal species were isolated from both litter and air [34]. The mean total fungal counts in shavings or wood chips from broiler and layer houses ranged between 10^2^ and 10^8^/g with many samples that exhibited variable counts for most of the species defining contrasted growth patterns [36, 40, 49]. Global densities were slightly lower than that of the final samples of litter [49]. Conidial populations in five broiler litters showed significant differences varying from 1.4 × 10^5^ to 7.8 × 10^5^/g [51]. The role of variations in pH and moisture content on fungal population densities remained controversial although localized damp and soiled areas under troughs or feeders might promote fungal development [26, 36, 40, 51]. In turkey farms of central Iowa, increased numbers of *Aspergillus* conidia correlated with higher counts of moulds in bedding suggesting that the species arose from the litter or that environmental conditions were favourable to growth and sporulation [35]. Lair Fulleringer et al. [44] reported very low and constant densities of *A. fumigatus* in fresh straw litter in a turkey house. Lovett [52] isolated a toxinogenic *A. fumigatus* from a poultry litter, which induced chick embryo death at 9 days. 

Cases of acute aspergillosis have been attributed to changes in litter management. Dyar et al. [17] incriminated the addition of a dry hardwood mixture that had been used for the treatment of moist places, to explain the increased mortality of 3.5-week-old turkeys due to *A. fumigatus* infection. The added litter was highly contaminated with 2.5 × 10^6^ cfu/g and contained at least 25 times more fungal organisms per gram than the original litter. Subsequent treatment of the bed with a fungistatic compound reduced both mould counts and mortality. In two broiler houses, the replacement of rice-hull beddings by *A. fumigatus-*contaminated sunflowers shell was associated with a severe aspergillosis. Removal of mouldy litter resulted in health improvement [16]. The direct application of feed on the litter and an average concentration of 1.3 × 10^4^ *Aspergillus *cfu/g of wood shavings were incriminated in an outbreak of aspergillosis affecting a broiler breeder flock [3]. The sporadic use of fresh sugarcane bagasse instead of traditional storage stacked bagasse was associated with up to 90% mortality in six flocks of young chickens on the Island of Barbados. The very high moisture of the fresh material seemed to be highly favourable for *A. fumigatus* growth and sporulation. No clinical case occurred after reintroduction of stored bagasse as litter [26].

### 2.4. Mycoflora of Poultry Feedstuffs

A wider variety of fungal genera were isolated from litter than from feed. Up to twelve fungal genera, with dominant *Aspergillus*, *Fusarium*, *Mucor*, and* Penicillium*, were isolated in feed with total densities varying from 7 × 10^2^ to 3.2 × 10^5^ cfu/g [40]. The species *A. fumigatus* yielded at a maximum of 2.3 cfu/g in turkey commercial feed [44]. 

### 2.5. *Aspergillus fumigates* Genotyping and Epizootiology

Due to its ubiquitous distribution combined with favourable environment in confinement buildings, all poultry flocks are virtually at risk of inhaling airborne conidia of* A. fumigatus* during the rearing or laying period [4]. Subsequent dispersal of small-sized conidia throughout the entire respiratory system is frequent resulting in isolation of the fungus from the lungs of healthy birds [34, 53, 54]. The impossibility to discriminate between clinical and environmental isolates indicates that every isolate is potentially pathogenic if it encounters a susceptible host [4, 33, 53]. Therefore, pertinent molecular tools are needed to clarify aspergillosis outbreaks in poultry sectors by determining the sources, the transmission modalities, and finally the colonization and infection patterns of fungal isolates. Many typing techniques have already been described for *A. fumigatus* and evaluated on isolates from human cases [55]: Random Amplified Polymorphic DNA (RAPD) [56], Restriction Enzyme Analysis (REA) [57], Restriction Fragment Length Polymorphism (RFLP) [58], Amplified Fragment Length Polymorphism (AFLP) [59], Microsatellite Length Polymorphism (MLP) [60, 61], Multilocus Sequence Typing (MLST) [62], and CSP typing [63]. Two different highly discriminant methods have been tested on avian isolates. Seventy-eight distinct genotypes were obtained by microsatellite typing of 65 clinical isolates and 23 environmental isolates [64]. Using two polymorphic microsatellite markers, Lair Fulleringer et al. [44, 54] demonstrated a very high polymorphism of *A. fumigatus* isolated either from the environment or from internal organs of both healthy and diseased animals in a turkey farm in France. Samples from birds, litter, feed, or air might share common genotypes. When entering the building, one-day-old turkeys harboured a unique genotype in their lungs whereas from one to six distinct profiles were evidenced in turkeys sacrificed during the rearing period [54]. The majority of the genotypes isolated from air and litter were observed only once (62% and 80%, resp.). The remaining genotypes were detected repeatedly with a maximum of persistence of 8 weeks. The chronology of genotypes suggested multiple sources of contamination and intense circulation of *A. fumigatus* isolates in the farm [44, 54]. Two turkeys with aspergillosis were infected by their own but distinct genotype [54]. In contrast, Olias et al. [53], performing genomic fingerprinting by microsatellite assay or sequencing of ITS-1 region on clinical isolates from stork chicks (*Ciconia ciconia*), proved polyclonal infections in all birds. Similar results were obtained on captive penguins with invasive aspergillosis. Isolates presenting distinct RAPD, STR, and enzyme activities patterns were recovered from different birds but not all [11]. However, interpretation of polyclonal infection of the lungs may be confusing until the role of each genotype in promoting infection is elucidated. A laser microdissection technique allowed direct capture of intralesional hyphae and subsequent genotyping [53, 65]. 

We recently developed a new typing method based on multiple-locus variable-number tandem repeat (VNTR) analysis (MLVA) for *A. fumigatus*. The combination of 10 VNTRs displayed high discriminatory power, stability, and reproducibility. We tested clinical and environmental isolates recovered from different poultry systems in China and France. Results revealed a clear clustering according to the geographic origin of the isolates rather than to their respective hosts [66].

The origin of the remarkable variability encountered within *A. fumigatus* remains uncertain. The recent discovery of a sexual stage could explain the presence of multiple genotypes. If proven in field conditions, the recombination may have significant epidemiological and pathological implications but may also question the clonality assumption [67].

## 3. Prerequisites to Avian Aspergillosis Onset

### 3.1. Virulence of *Aspergillus fumigates*

Virulence represents the ability of a pathogen to invade the host, overcome its natural defences, and proliferate subsequently in the organism. When sensing a favourable environment, *A. fumigatus* conidia germinate and concurrently produce enzymes that degrade organic materials into nutrients for further assimilation. The fungus secretes various enzymes like proteases and toxic secondary metabolites [32, 33, 68]. Gliotoxin is a highly immunosuppressive mycotoxin produced by various isolates of *A. fumigatus*. Concentrations exceeding 20 *μ*g/g and 70 *μ*g/g have been detected in poultry feedstuffs [69] and in tissues obtained from turkeys with airsacculitis [70], respectively. Turkey blood peripheral lymphocytes, when exposed to high levels of gliotoxin, either died or exhibited a lower lymphoblastogenic response [71]. Considerable amounts of gliotoxin were found in lungs of turkeys just four days after experimental inoculation of *A. fumigatus *[72]. However, the distinction of true virulence factors [33] remains uncertain because either environmental or clinical isolates seem to be able to induce an aspergillosis in susceptible hosts [64, 68]. In experimental conditions, intra-air sac inoculation of turkeys with mammalian, avian, or environmental *A. fumigatus* isolates induced mortality and lesions in all groups but one [73]. The species *A. fumigatus* might express virulence without requiring specific and unique fungal determinants. Indeed, the analysis of the genome of *A. fumigatus* suggests that its primary ecological niche is in plants and that opportunistic infections of animal hosts are a dead end for this fungal species [4, 68].

### 3.2. Interactions of *Aspergillus fumigates* with the Avian Respiratory System

Both host and fungus characteristics explain the particular susceptibility of birds to *A. fumigatus* infection. Aspergillosis is primarily an infection of the respiratory tract [4, 74]. Birds placed in environments contaminated with aerosolized conidia may show significant pathology after only a short duration of exposure. Anatomy and physiology of the avian lung-air sac system are strikingly different from that of the bronchoalveolar lung of mammals. Nine air sacs function as bellows to move air through the lungs' gas-exchange surface [74–76]. Upper respiratory clearance mechanisms rely on mucous-covered epithelial cells possessing cilia and lining the trachea, the primary bronchi, and the roots of the secondary bronchi [74, 76]. The epithelium of the upper airway presents also a highly lytic activity [77]. When unanesthetized chickens [78] or anesthetized pigeons [79] were exposed to aerosolized populations of various size fluorescent microspheres, particles with a diameter of 3 *μ*m or less were found throughout the respiratory tract. On the contrary, bigger spheres were confined to upper airways where mucociliary-dependent clearance might occur [78, 79]. Therefore, *A. fumigatus* conidia are small enough, 2-3 *μ*m in diameter, to bypass initial physical barriers and disseminate deeply in the respiratory system. The gas pathway through lungs accounts for the susceptibility of the caudal air sacs to pathogen infections including mycosis, compared to the cranial air sacs [74, 76]. The larger diameter of *A. flavus* conidia (3.5–4.5 *μ*m) [4] may explain their lower pathogenicity when compared to *A. fumigatus* in experimental infections [80]. In a recent study [81], the effects of *Aspergillus* conidia on human respiratory cell apoptosis was evaluated. *A. fumigatus* and *A. flavus* conidia inhibited cellular apoptosis, while *A. nidulans*, *A. niger*, and *A. oryzae* conidia did not. However, there were no differences in the inhibition of apoptosis by *A. fumigatus* conidia from either human, avian, or environmental isolates [81]. 

The avian lung-associated immune system includes a bronchus-associated lymphoid tissue (BALT) localized at the junctions of primary and secondary bronchi and at the ostia to the air sacs, an interstitial immune system combining lymphocytes and macrophages and a phagocyte system [76, 82]. The latter should provide an immediate front line defence of the extensive gas-exchange surface area as observed in mammals [75, 77]. Lavages of the normal steady-state avian respiratory system yield very few resident phagocytes that gather in clusters at the entrance to air capillaries [82, 83]. Avian air sacs are particularly prone to contamination because they are submitted to an airflow that favours particle deposition. They have no available macrophages to remove foreign items and have an epithelial surface nearly devoid of a mucociliary transport mechanism [75]. In contrast, access to blood-gas barrier tissue is protected by an extensive phagocytic epithelium [77]. Furthermore, although rather refractory to elicitation by inert stimulants like nonviable *A. fumigatus* conidia [84], the avian respiratory system responds efficiently to invasion by pathogens with a rapid influx of heterophils and macrophages from the subepithelial compartment and pulmonary vasculature [77, 83]. During the acute phase response, kinetics of migration suggests that heterophils egress earlier and in larger number from the tissues to the lumen of the lungs. Those primary polymorphonuclear leukocytes are vital cellular components of innate immunity and function by killing the pathogens following phagocytosis [84]. Classical avian macrophage properties include chemotaxis, phagocytosis, pathogen elimination, and cytokine production [85]. Many macrophages of turkeys exposed to *A. fumigatus* by aerosol 45 min earlier had conidia attached to them or had ingested one or more conidia [86]. The ability of birds to respond to fungal antigens by organising a good and lasting cell-mediated response could be a determinant in infection resolution which means that one of the key events in the establishment of aspergillosis may be the resistance to phagocytosis and its slow killing *in vivo* [33].

Finally, if the avian respiratory system appears to lack any of the clearance mechanisms found in mammals, an effective resolution of infection largely depends on a precocious and strong recruitment of activated phagocytes [75, 83].

## 4. Natural Avian Aspergillosis

### 4.1. Clinical Signs

Acute aspergillosis may include a variety of nonspecific clinical signs: anorexia, lethargy, ruffled feathers, respiratory signs, polydipsia, polyuria, stunting, or sudden death. In chicks, contaminated *in ovo* or during hatching, the disease, commonly known as brooder pneumonia, is highly fatal in the first ten days of life and results in a major respiratory distress [2, 4, 87, 88]. Two outbreaks of omphalitis where the primary cause was *A. fumigatus* have been investigated in young turkeys [18]. In poultry farms, mortality rate may rise slightly [14] or increases suddenly, peaks during a few days, and then returns to initial state [2, 3, 15–17, 89]. Respiratory signs include dyspnoea, gasping, hyperpnoea with panting, nonproductive coughing, wheezing, cyanosis [3, 14, 15, 17, 21], and sometimes nasal discharge [19].

In the chronic form, dyspnoea, depression, dehydration, and emaciation are described. Nervous system involvement causes ataxia, tremor, opisthotonos, lateral recumbency, torticollis, seizures, convulsions, lameness, and hind limb paresis [3, 14, 17, 89]. Occurrence of nervous and ophthalmic troubles one week after an acute episode of aspergillosis has been reported in a turkey flock [17]. Cloudiness of the eye with severe conjunctivitis and turbid discharge were associated with paralysis in broiler breeders [3].

*A. fumigatus* can colonize skin and surgical wounds [7] as observed in caponized cockerels [90] and induce necrotic granulomatous dermatitis [91] or even systemic aspergillosis.

### 4.2. Gross Lesions

**Figure 1:**
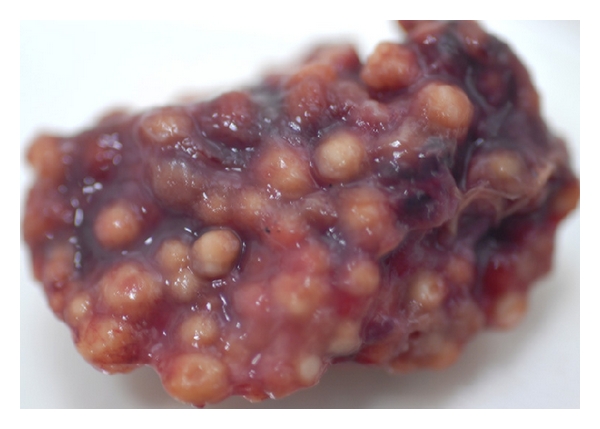
Numerous nodules in the lung of a duck with acute aspergillosis.

The severity and the degree of development of the disease determine both morphology and extension of macroscopic lesions. Extensive involvement of the respiratory tract can occur before clinical signs are apparent. Typically, lesions consist of white to yellowish granulomas ranging from miliary (<1 mm in diameter) to large roughly spherical granulomatous nodules (>2 cm) involving serosae and parenchyma of one [15] or multiple organs. Single or multiple necrotic areas are visible on cut surfaces. The primary location of lesions is the air sacs and lungs although oesophagus, proventriculus, gizzard, small intestine, liver, kidney, spleen, skin, trachea, peritoneum, brain, eye, muscle, or heart may be involved [1, 3, 5, 14, 19, 21, 22, 92]. Lung parenchyma is either consolidated or has focal granulomas of varied size [2, 23, 93] (Figure 1). When coalescing in air sacs, these masses form cheesy caseous plaques covering the thickened membranes and even obstructing the entire lumina where fungal sporulation may occur as evidenced by a grey-greenish velvet [3, 14, 19, 22, 92, 93]. 

Nonulcerative or mildly ulcerative keratitis was reported in a turkey flock [17]. Broiler pullets presented periorbital and eyelid swelling with cheesy yellow exudates in the conjunctival sac [3]. Circumscribed white to greyish areas were observed in the cerebellum of broiler breeders [3] and turkeys [89]. Ribs of ostriches [2], sternum of broiler breeders [5], yolk sacs of poults [18], and hip joints of turkeys [20] constituted unusual locations of aspergillosis lesions. 

### 4.3. Histopathology

Haematoxylin-eosin stain is often augmented with periodic acid-Schiff, Grocott, and Gomori's methenamine silver stains in order to display fungal elements in embedded tissue sections [5, 14, 16, 18, 92]. The fluorescent optical brightener blankophor proves to be a valuable tool for demonstrating *Aspergillus *sp. hyphae [65].

Based on histopathological differences, Cacciuttolo et al. [92] distinguish a deep nodular form and a superficial diffuse form of aspergillosis. A well-organised granulomatous reaction develops in nonaerated parenchyma whereas a superficial diffuse form, containing fungal elements and a nonencapsulated pyogranulomatous reaction, predominates in serosae and lungs [89]. Organised granulomas are clearly encapsulated by an outer thick fibrous layer [22, 23] whereas pyogranulomas lack clear borders. The first feature corresponding to late granuloma may also be an important sign of the chronic form of aspergillosis, especially in adults [21, 22, 93]. Pyogranulomas organization presents a centre with variable amounts of septate, dichotomously branching hyphae surrounded by a palisade of radially arranged foreign body giant cells, macrophages, heterophils, and lymphocytes. Phagocytized fungal elements are regularly observed in the eosinophilic cytoplasm of multinucleated cells. Lymphocytes may infiltrate the margins of the granuloma [1, 3, 5, 16, 18, 19, 22, 89, 92]. Progressive inflammation in lungs resulting in small granuloma coalescence induces more extensive lesions and may lead to parabronchial obliteration with necrotic eosinophilic material containing erythrocytes, degenerated heterophils, and exfoliated epithelial cells [5, 93]. Numerous conidiophores and free spores appear in granulomas that open to the air spaces of the respiratory tract [22]. 

Typical granulomatous reactions associated or not with fungal elements have been observed in brain [17, 18], conjunctiva [93], liver, spleen, gizzard, small intestine [22], hip joints [20], and trachea [15].

### 4.4. Diagnosis

*Antemortem* diagnosis of aspergillosis, particularly in chronic cases, remains a challenge. Exotic pets may benefit from cumulative diagnostic tests including biochemistry, haematology, radiography, laparoscopy, or endoscopy [94] that are not available in a poultry context. 

Aspergillosis should be strongly suspected when debilitated birds with respiratory distress do not respond to antibiotic treatment [4, 20] and when careful history reveals the presence of underlying environmental or immunosuppressive factors. Definitive diagnosis is based on the isolation of *A. fumigatus* by culture or by the detection of the organism during histological examination [4]. It is fundamental to keep in mind that many birds can host *Aspergillus* conidia in the respiratory system leading to a dormant infection without clinical symptoms or macroscopic lesions [92]. Furthermore, *A. fumigatus* may not be the primary cause [14] or may be associated with either other pathogens like *A. niger *[2, 3] or *A. flavus* [89] or a concurrent nonfungal disease [16, 17, 20].

Immunohistochemistry, with monoclonal or polyclonal antibodies, is a powerful and accurate tool to identify *A. fumigatus *in lesions [18, 22, 89, 93].

Serological tests have not been validated in poultry and are not currently used in farms to investigate aspergillosis outbreaks [4, 95].

#### 4.4.1. Intervention Strategies

Although numerous antifungal protocols have been proposed to cure birds with aspergillosis [96], treatment of the disease in poultry farms is virtually impossible. No vaccine is available. Specific biosecurity measures against *Aspergillus* contamination rely primarily on prevention. Dust and mouldy litter or feed should be avoided. A good litter management combined with daily assessment of its quality throughout the lifetime of the flock is the key to prevention of the disease. Bed, like feeders, should be kept dry, nondusty, and clean in order to limit fungal development [4, 50]. Control of relative humidity through appropriate ventilation should be verified to prevent wet litter [4, 35]. Sporadic or repeated antifungal treatment may be useful in order to control environmental contamination. Spraying of fungistatic agents like thiabendazole [39], nystatin, or copper sulphate [17] contributed to decreased fungal contamination of beddings. Enilconazole may be sprayed, fogged, or nebulised to decontaminate surfaces or indoor volume [21, 88]. Finally, effects of stressors like beak trimming and high stocking densities should be minimized [3].

## 5. What Can We Learn from Aspergillosis Avian Models?

Animal models are conceived to improve our understanding of pathogens virulence, disease pathogenesis, and therapy feasibility [97]. Because of unique anatomical and physiological features of the avian respiratory system [74], the use of birds to study avian aspergillosis as a global entity is required. More restricted models, like embryonated eggs, have been developed to investigate *A. fumigatus *development *in ovo* [87, 98, 99] to evaluate virulence of isolates in arthropods [100]. 

### 5.1. Avian Models of Aspergillosis

Numerous experiments, which differed largely in both *A. fumigatus *conidial concentrations and inoculation routes, have been reported on varied avian species [38, 80, 101–110] (Table 1). In some surveys, birds were concurrently challenged with *A. flavus *conidia [80, 101]. The purpose of intratracheal, intrapulmonary, or intra-air sac injections [109] was to induce a primary respiratory disease. These inoculation routes allowed the delivery of consistent numbers of conidia between individual birds [105]. Nevertheless all of these routes bypassed a more or less important part of the upper airways and their associated defence mechanisms, especially when thoracic or abdominal air sacs were chosen [74]. The basic pattern of early pneumonia may be modified. Following a unique air sac inoculation, lesions are generally confined to the ipsilateral lung and air sacs. However, lesions are mostly compatible with those seen in field cases in which airsacculitis and pneumonia are the predominant findings [105, 111]. Conidial nebulisation aims to mimic natural conditions of contamination but requires a strict standardisation of procedures. Variable parameters include time exposure (5 to 60 min), nebulisation chamber volume, conidial concentration, and inoculum presentation [80, 101–103, 112]. Pulverized dry conidia compared to wet conidial aerosols yielded better results in terms of mortality and morbidity suggesting that the size of the droplets including conidia could be a drawback [103]. However, the dosage of a dry inoculum lacks precision and its manipulation is delicate. Aerosol infection allows a large number of conidia in the lung as measured by the number of conidia/g of lung immediately after exposure. A loading charge of 5 × 10^5^ viable conidia/g of lung killed 50% of 3-week-old turkeys [80], whereas no mortality was reported in one-day-old chickens exhibiting 3 × 10^4^ spores/g just after aerosolisation [101]. 

Experimental immunosuppression was used in order to induce an aspergillosis in less susceptible species or to reduce the variability of diseased birds response. The protocols consisted of repeated dexamethasone injections (2 to 5 mg/kg) in pigeons [109], chickens (see [113]; data not shown), and turkeys [106]. Genotyping of isolates recovered from internal organs allowed to verify that experimental infection resulted from the inoculated *A. fumigatus* strain [109].

In most cases, experimental aspergillosis is a hyperacute infection obtained by administering a very high concentration of conidia at a single time point. When observed, deaths and morbidity occur generally between day 1 and day 14 [73, 80, 103, 106, 108, 114]. From 7 days after-inoculation, surviving animals may develop chronic lesions [112]. These models reproduce a pulmonary aspergillosis with clinical signs and internal lesions. In some cases, extensive intraocular lesions [80] or neurological symptoms with associated brain granulomas [112] have been described after aerosol exposure. 

### 5.2. Pathogenesis

Sequential necropsies at various times after inoculation allow observations on the pathogenesis of the disease and the kinetics of internal lesions development [80, 101, 104–106, 112]. Immediately after aerosol exposure and up to 24 h later, *A. fumigatus* conidia were detected in circulating blood of 3-week-old turkeys. In contrast with mycological cultures of lung tissue, the proportion of *Aspergillus-*positive cultures from liver and brain decreased with time. Numerous conidia could be observed in many macrophages harvested by lung lavages. Therefore, brain or eye lesions due to *A. fumigatus* may follow rapid haematogenous dissemination of conidia mediated by macrophage migration from the respiratory system [86, 112, 115].

Air sac membranes were slightly opaque 24 h after air sac inoculation and scattered with miliary white foci. As fulminant airsacculitis evolved, the severity of macroscopic lesions increased rapidly, with progressive thickening, vascularisation, and opacification. Concurrently, 1–5 mm granulomas developed and tended to coalesce in plaques. Early lesions in lungs consisted of marginal oedema, progressive consolidation, and the formation of small white nodules [80, 101, 104–106, 112].

In the first hours following infectious challenge, clear oedema, extensive epithelial alterations, and massive infiltration of heterophils, macrophages, and lesser lymphocytes occurred in both lungs and air sacs. Scarce swollen and germinating conidia were observed in inflammatory exudates, in areas of necrosis and aggregates of epithelial macrophages or multinucleated giant cells. *A. fumigatus *seemed to infect the air sac membrane interstitium rather than its surface leading to a severe airsacculitis within 24 h. Granulomas with heterophilic or necrotic centres and fungal elements were reported as soon as 24 h after air sac inoculation in 9- and 19-week-old turkeys [105], but not in one-day-old turkeys [104], where diffuse inflammation predominated till 48 h. Chronologically, multifocal inflammatory response was gained in cellularity with marked mixed cellular infiltrates and granulomatous reaction in respiratory tissues. Circumscribed pyogranulomas developed with central eosinophilic necrosis restricting short hyphae and conidia surrounded by intact heterophils, epithelioid, and foreign body giant cells [80, 104, 105]. Beyond 6 days after exposure, inflammatory and necrotic foci seemed to regress in surviving birds exhibiting well-organized granulomas encapsulated by a thick layer of fibrous tissue [80, 112]. Destruction of *A. fumigatus* occurred as attested by fungal elements observed in multinucleate giant cells and the regular diminution of positive cultures from tested organs [80, 101, 104, 105].

**Figure 2:**
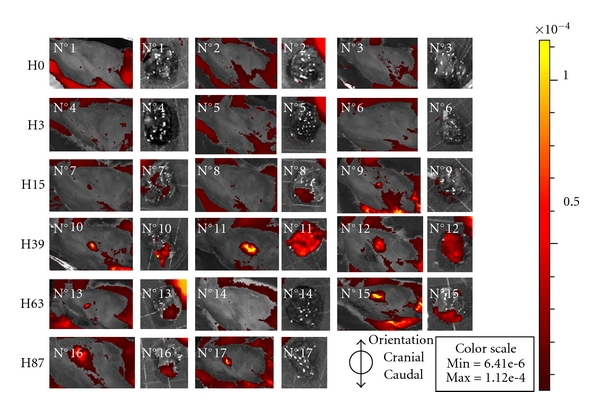
Fluorescence intensity emitted by injected DsRed conidia detected by imaging on both whole body and left isolated lung at different times (in hours) after inoculation (three chickens per time).

In order to improve our current understanding of pathogenesis, crucial first steps of host-pathogen interaction should be carefully monitored. Bioluminescence or fluorescence imaging techniques using engineered *A. fumigatus *strains made it possible to follow the progression of aspergillosis *in vivo* in real time as demonstrated in a mammal model [116]. We recently evaluated the possibility of following the localisation and development of fluorescent *A. fumigatus* conidia in the respiratory tract of chickens by imaging. Air sac inoculation was performed on 18 animals with 10^8^ conidia expressing the DsRed protein [117]. Thirteen control birds were inoculated with a suspension of 10^8^ red fluorescent microspheres (Merck). Groups of 2 to 3 birds were slaughtered at 87 h, 63 h, 39 h, 15 h, 3 h, and less than 5 min after inoculation. The chicks were examined by fluorescence imaging in the red spectrum in order to limit absorption and autofluorescence (IVIS Spectrum system, Caliper USA) then autopsied. Both the type and the extent of the lesions were noted, and the left lung was removed and imaged. The amount of each fluorescent signal was quantified for the whole animal and the isolated lung. The results showed a progressive development of the signal in the respiratory tract, which was correlated with the production of fluorescent hyphae: 5 min and 3 h after inoculation, the DsRed fluorescent signal was weak for both the entire animal and isolated lungs; 15 h after inoculation, a fluorescent signal was more visible in birds at the injection point and in the caudal part of the lungs; 39 h after inoculation, fluorescence was intense for the 3 chicks; 63 h after inoculation, the signal intensity decreased but remained stable until 87 h (Figure 2). There was no correlation between the numeration of viable conidia in the lungs (maximal counts between 5 min and 15 h) and signal efficiency on one hand and the extension of pulmonary lesions on the other hand. The emitting surface (fluorescent hyphae) and the macroscopic pulmonary lesions were very closely superimposed (data not shown). Individual variability of results following red microsphere inoculation of a small number of chickens (data not shown) may be due to random dispersion of microspheres in the lungs and air sacs after several respiration cycles. The higher repeatability of results in chicks inoculated with labelled conidia could be explained by the early germination of conidia and the subsequent development of the hyphae surface which increased signal efficiency. Conidia labelled with fluorochromes stimulated by higher wavelengths should allow a more precise followup of infection by reducing inoculum concentration and optimizing the signal quality. 

### 5.3. Biological Markers

**Figure 3:**
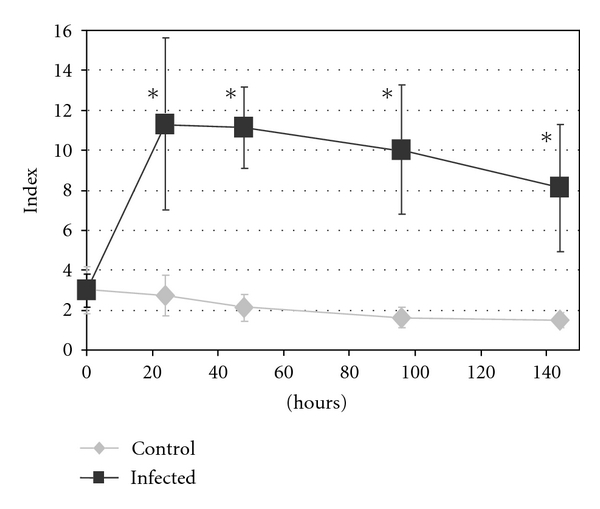
Mean concentration of seric galactomannan in control and experimentally infected turkeys. Index = optical density of the sample/mean optical density of two threshold samples (1 ng/mL of galactomannan) provided in the Platelia kit. *Significant difference (*P* < .05).

Relevant serological markers and reliable diagnostic tools allowing improved aspergillosis diagnosis or experimental infection monitoring are still lacking in birds. Research strategies may include evaluation of reported markers and identification of new putative candidates. As with previous experiments, we tried to identify potential biological markers in turkey poults experimentally infected by *A. fumigatus *[106]. Eighteen immunocompromised birds were infected by inoculation within the left posterior thoracic air sac with a suspension containing 10^8^ *A. fumigatus* conidia. Birds in the control group (*n* = 18) were similarly immunosuppressed but not exposed to the conidial suspension. Blood was sampled, and necropsies were performed in birds sequentially sacrificed at days 0, 1, 2, 4, and 6 after inoculation. Circulating serum galactomannan, a major parietal antigen of *A. fumigatus *[32, 118], was detected using Platelia *Aspergillus* kit. Galactomannan concentrations of infected birds increased markedly 24 h after inoculation (mean index = 11.5) then decreased slowly until 144 h (mean index = 8.0) (Figure 3). In control animals, galactomannan concentration remained stable and significantly lower than in infected poults throughout the experiment. We demonstrated that circulating galactomannan should be considered as an interesting biomarker in experimental context, which occurred earlier than clinical signs and lesions in experimentally infected turkeys. Since it is produced by active hyphae only, galactomannan concentrations could allow the monitoring of the first steps of fungal development in tissues [32]. However, the ubiquitous presence of galactomannan in many poultry feeds may lead to false positives and reduces its efficacy in field conditions. 

**Figure 4:**
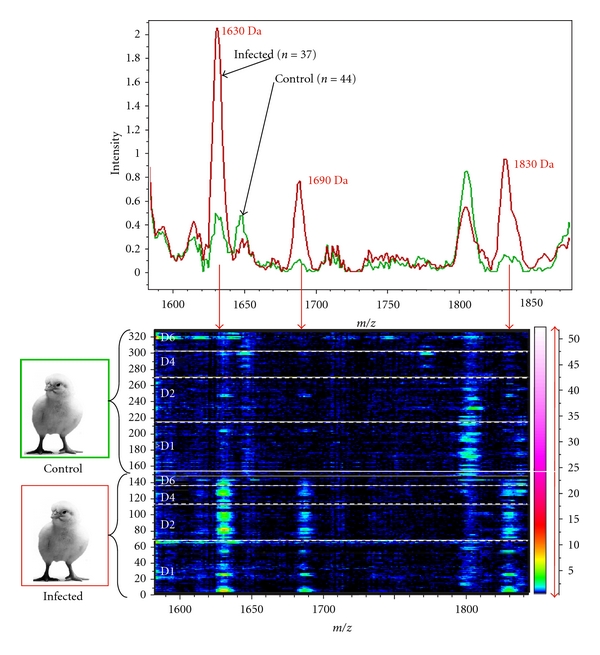
Example of mean spectrum obtained by matrix-assisted laser desorption ionization time of flight mass spectrometry (WCX) from seric samples of infected and control turkeys.

In order to compare seric proteins from infected and noninfected birds, we used matrix-assisted laser desorption ionization time of flight mass spectrometry (MALDI-TOF MS). Our preliminary data showed that the levels of 13 proteins or peptides were significantly altered in infected turkeys compared with controls. Furthermore, the concentration of some of these markers increased from day 1 to day 6 in birds challenged with* A. fumigatus *conidia (Figure 4). Subsequent characterization of peptide candidates by tandem mass spectrometric fragmentation and *de novo* protein sequencing, MALDI-TOF/TOF-MS, is needed to evaluate their potential as valuable markers. Seric peak patterns differed strikingly in turkey and chicken models (data not shown).

### 5.4. Susceptibility to *Aspergillus fumigates* Infection and Genetic Resistance

Both field data [115] and experimental results [114] clearly demonstrated a higher susceptibility of turkeys and quails to *A. fumigatus *infection when compared to chickens. 

At the species level, recent genetic approaches to immune modulation and disease resistance via lines of targeted selection could advantageously complement medical treatments and improved management in poultry farms [119]. For example, differences among commercial broiler lines in macrophage effector function such as phagocytosis suggest the opportunity to exploit macrophage-based immunocompetence in selection programs [120]. Exposure of three distinct strains to increasing numbers of aerosolized conidia/m^3^ leads to a greater mortality of White Leghorn than Athens Canadian or Vantress × Arbor Acres chicks. Interestingly, the percentage of survivors with lesions remained constantly lower in the first strain whatever the inoculum concentration [37]. We recently compared three lines of White Leghorn selected for high antibody, high cell-mediated immune response, and high phagocytic activity [119] in an experimental model of aspergillosis. Our results established a similar ability of all genetic lines to eliminate the fungus 7 days after intrasac inoculation (data not shown). A greater proportion of small Beltsville white turkeys developed pneumonia and airsacculitis following experimental challenge as compared with broad-breasted white turkeys [107].

### 5.5. Acquired Immunity and Treatments

The role of acquired immunity in aspergillosis resolution still remains unclear. A previous nonfatal challenge with *A. fumigatus *did not protect turkeys against a second inoculation and even worsened airsacculitis severity [107]. Transfer of activated splenocytes from convalescent 12-to-14-week-old Beltsville small white turkeys to naive birds did not confer any protection against experimental infection to the latter [121]. A culture filtrate vaccine, a conidial vaccine, a mycelial vaccine, and 2 germling vaccines were compared in different trials for their protective efficiency against *A. fumigatus* infection in poults, with limited results [122, 123]. This underlines the actual importance of animal models in therapeutic protocols evaluation [102, 103, 124].

## 6. Concluding Remarks

The conditions which allow *A. fumigatus* to provoke infection in only some of the poultry in a rearing unit remain unclear. The outcome of the disease probably depends on the first steps of the innate immune response, which relies on the influx of macrophages and heterophilic granulocytes. Deciphering interactions between the different active effector cells is far from being as well defined as it is in mammals. In chickens and turkeys, there is a constant increase in knowledge concerning the identification of cytokines, target membrane receptors and the immuno-modulation mechanisms. Another pathway, which could be studied, integrates the concurrent effects of the different biotic and abiotic components present in bio-aerosols on the respiratory and immune systems of birds in farms.

## Figures and Tables

**Table 1:** Models of avian aspergillosis.

Species	Age^a^	Experiment duration	Immuno-suppression^b^	Inoculation route^c^	Inoculum^d^	Mortality	References
Chicken (*Gallus gallus*)	1-day-old	21 days	N	L	ND	84%	[38]
Chicken	1-day-old	30 days	N	wAER	3.16 × 10^7^	0%	[101]
Chicken	1-day-old	42 days	N	dAER	5 mg	6.7%	[102]
					10 mg	9.9%	
					21 mg	17%	
					42 mg	37.8%	
					85 mg	53.6%	
					170 mg	83.5%	
					340 mg	93.3%	
Chicken	1-day-old	25 days	N	dAER	500 mg	100%	[103]
Turkey (*Meleagris gallopavo*)	1-day-old	7 days	N	IAS	10^6^	0%	[104]
Turkey	9-week-old	4 days	N		5 × 10^7^	0%	[105]
	18-week-old					0%	
Turkey	6-week-old	6 days	Y	IAS	10^8^	16%	[106]
Turkey	10-week-old	42 days	N	IAS	5 × 10^6^	0%	[107]
Turkey	3-week-old	56 days	N	dAER	5.18 × 10^8^	33%	[80]
					5.18 × 10^9^	55%	
Quails (*Coturnix japonica*)	2-week-old	42 days	N	IT	1.2 × 10^7^	20%	[108]
Pigeon (*Columba livia*)	4 to 5-week-old	7 days	N	IT	2 × 10^7^	25%	[109]
			Y	IP		100%	
			N			25%	
			Y	IAS		100%	
			N			100%	
Starling (*Sturnus vulgaris*)	ND	6 days	N	IT	1.35 × 10^6^	100%	[110]

^a^At inoculation time; ^b^dexamethasone injections (Y: yes, N: no); ^c^wAER: wet aerosol; dAER: dry aerosol; L: contact with a contaminated litter; IT: intratracheal; IP: intrapulmonary; IAS: intra-air sac; ^d^numbers of conidia/bird except for aerosol (total dispersed inoculum); ND: nondetermined.
